# Screw fixation after tripe pelvic osteotomy is reliable: changes of acetabular correction are rare and do not correlate with risk factors

**DOI:** 10.1186/s13018-023-04186-6

**Published:** 2023-09-21

**Authors:** Daniel Dornacher, Maximilian Kelsch, Mirco Sgroi, Heiko Reichel, Bernd Lutz

**Affiliations:** Department of Orthopedics, University of Ulm, Oberer Eselsberg 45, 89081 Ulm, Germany

**Keywords:** Triple pelvic osteotomy, Change of acetabular correction, Hip joint preservation surgery, Developmental dysplasia of the hip

## Abstract

**Purpose:**

The aim of this examination was to assess whether there is a change of acetabular correction after triple pelvic osteotomy (TPO) and if so, whether there is a correlation with patient-specific risk factors or with certain periods in the postoperative course.

**Methods:**

A consecutive series of 241 TPO was reviewed retrospectively. The close-meshed radiographic follow-up of the first 12 weeks comprised pelvic radiographs performed immediately after the procedure, 5 days, 6 and 12 weeks after TPO. Three observers measured the lateral center edge angle, acetabular index and the craniocaudal offset of the pubic osteotomy. Patient-specific risk factors (e. g. age, gender, body mass index, nicotine abuse) and certain periods in the postoperative course were correlated with a change of acetabular correction.

**Results:**

After application of the exclusion criteria, 225 hips were available for further examination. Intraclass correlation coefficient resulted in predominantly excellent agreement between the measurements of the three observers (0.74–0.91). In 27 cases (12%), the three observers agreed on a change of acetabular correction. In 18 cases (8%), there was a slight change, in 9 cases (4%), a relevant change. The latter entailed consequences in the postoperative aftercare. General equation estimation did not show any correlation between a change of acetabular correction and patient-specific risk factors or certain periods in the postoperative course (*p* = 0.79–0.99).

**Conclusion:**

Every once treated hip should be followed-up with the same attention, irrespective of the apparent risk profile. There is no rationale to skip a radiographic follow-up in the first 12 weeks after TPO.

## Introduction

In corrective osteotomies around the hip, there is a broad consensus that a precise acetabular reorientation is the key for the longevity of the natural joint. Looking at the surgical treatment of developmental dysplasia of the hips in adolescents and adults, it has been shown that acetabular under- and in particular overcorrection can lead to an unfavorable outcome [1–3]. In the recent years, a more comprehensive understanding has been developed for the radiographic analysis of acetabular malorientation [4, 5]. After deformity analysis, a set of radiographic parameters enables the surgeon to plan a physiological acetabular orientation. Finally, the surgeon has to transfer this knowledge to the operating room. During pelvic osteotomy, the acetabular reorientation is usually guided reliably by intraoperative fluoroscopy or radiography [6]. Knowing that in the vast majority of the cases, the once set acetabular orientation remains the same throughout the process of osteotomy consolidation; in the day-by-day clinical practice, the impression of a slight change of acetabular correction emerges every now and then. While a slight change of acetabular correction might be acceptable, a major change or loss of correction could alter acetabular parameters toward unphysiological values.

The goals of this examination were: (1) to analyze the radiographic follow-up of the first 12 weeks in a consecutive series of triple pelvic osteotomies (TPO) performed in our institution, with a particular focus on a change of acetabular correction. (2) to assess whether patient-specific risk factors or certain periods in the postoperative course correlate with a change of acetabular correction.

## Material and methods

**Fig. 1:**
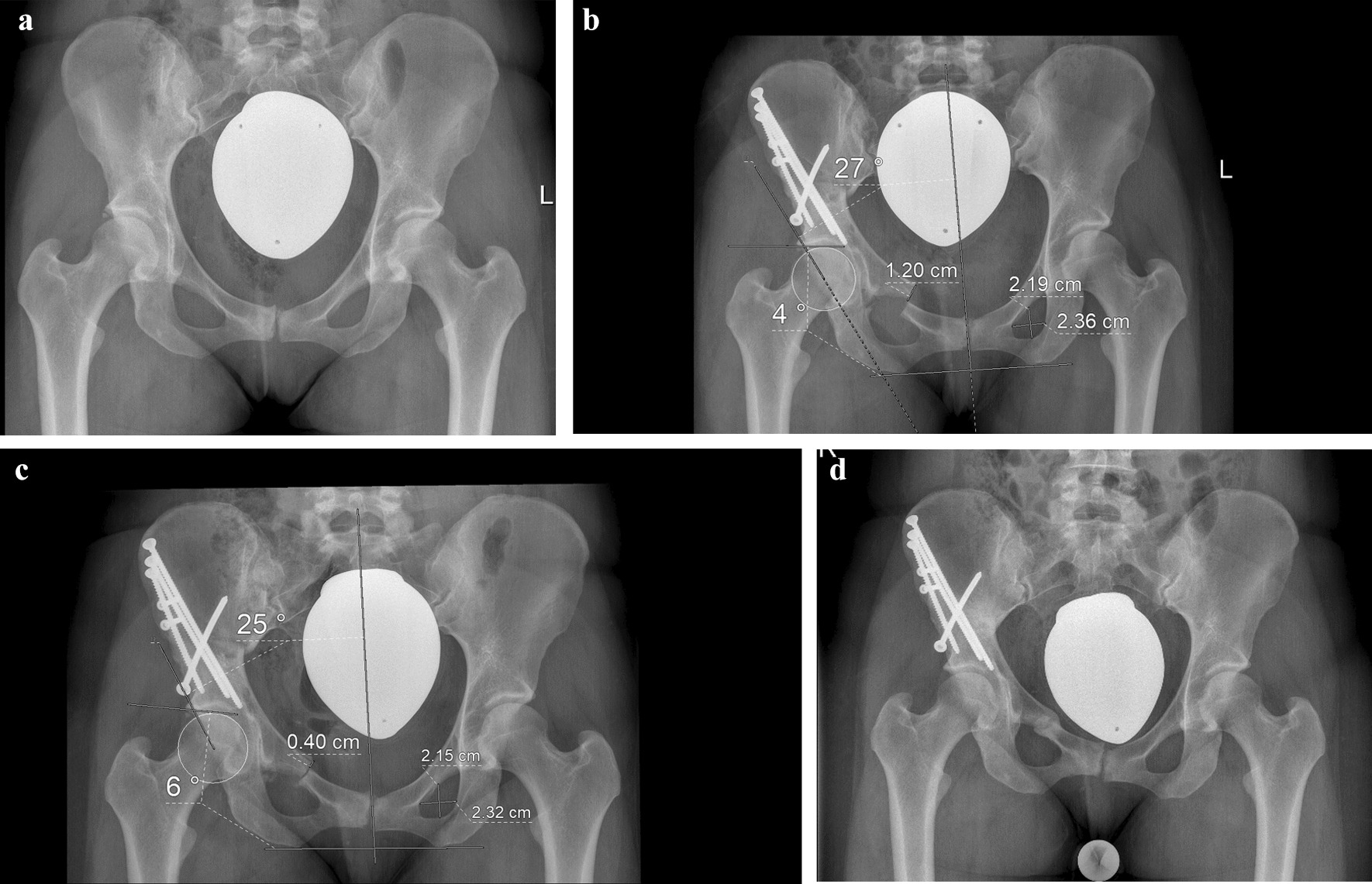
**a** Preoperative pelvic radiograph of a 29-year-old female with a BMI of 21.5 kg/m^2^, non-smoker, no comorbidities, clinical course free of accidents. **b** The image was performed 5 days after TPO, representing the first radiographic follow-up. The measurements revealed a LCEA of 27°, AI of 4° and a pubic osteotomy offset of 12 mm. **c** The second radiographic follow-up after mobilization and inpatient rehabilitation treatment, performed 6 weeks after TPO. Compared with the first follow-up, the width-height-relation of the left obturator foramen remained unchanged (1.08, resp.). Therefore, a comparable pelvic orientation was assumed. Most apparently, the pubic osteotomy offset (4 mm) changed in comparison with (**a**). LCEA was measured 25° and AI 6°. In the further follow-up, the parameters did not show any further discrepancies. **d** This image was not subject to this examination since it was performed ten months after TPO. The image shows no further change of acetabular correction and demonstrates an undisturbed consolidation of the osteotomies

A consecutive series of 241 TPO was reviewed retrospectively. All pelvic osteotomies were performed from January 2015 to December 2019 in our orthopedic department in total of 206 patients (178 female, 28 male) (for demographics in detail please see Table 1). The patients were predominantly referred to our outpatient department with the diagnosis of a symptomatic hip dysplasia. The TPO was performed in a highly standardized fashion by two surgeons. The acetabular fragment was fixed with 4.5 mm fully threaded steel screws, predominantly in a specific pattern using four screws: The objective was to spread three screws widely over the osteotomy and to place one screw perpendicularly (Fig. 1). In a recently published finite element analysis, it has been shown that the predominantly used fixation pattern with widely spread screws over the osteotomy and a perpendicular fixation generated an improved stability [7]. In the first few days after surgery (period one: mobilization), the patients were mobilized by experienced physiotherapists, using underarm sticks. Partial weight-bearing was allowed (20 Kilograms (kg)) for the first 6 weeks after surgery (period two: partial weight-bearing). After 1 week of inpatient treatment, almost all patients were referred directly to our in-house rehabilitation center in order to receive 3 weeks of specific rehabilitation measures, followed by outpatient physiotherapy on prescription. When the radiographic follow-up 6 weeks after surgery did not reveal any relevant changes in acetabular correction, partial weight-bearing was increased step-by-step (10 kg per week) until full weight-bearing was reached (period three: increasing weight-bearing). Finally, a further radiographic follow-up was scheduled 12 weeks after surgery.

**Table 1:** Demographics of the included cases

Included cases (n)	225	GEE			
		Parameter estimates	Lower limit	Upper limit	*p*
Gender	Females 195 (86.7%) Males 30 (13.3%)	0.620	0.001	2.795	0.878
Age to the time of surgery (years; mean, SD)	26.3 ± 8.5	1.019	0.770	1.349	0.890
Body weight (kg; mean, SD)	69.3 ± 16.7	0.998	0.866	1.150	0.982
Body height (cm; mean, SD)	168 ± 9	0.618	0.000	2.652	0.971
Body mass index (BMI) (kg/m^2^)	24.5 ± 4.9	0.998	0.620	1.609	0.996
Nicotine abuse (n)	63 (28%)	0.695	0.002	2.103	0.900
ex-smoker (n)	3 (1.3%)	0.629	0.002	1.520	0.868
American Association of Anesthesiologists (ASA) classification (n)	I: 119: II: 98; III: 8; IV: 0	0.792	0.010	5.748	0.915
Wound healing disorders (n)	7 (3.1%)	3.346	0.000	3.127	0.795
History of trauma or fall (n)	10 (4.4%)	0.799	0.000	2.324	0.972
LCEA preoperative (°)	16.7 ± 9.2	1.021	0.776	1.284	0.876
AI preoperative (°)	13.9 ± 8.3	0.987	0.758	1.284	0.923
Variation of predominantly used fixation screw pattern (n)	75 (33.3%)	1,466	0.011	1.835	0.876

The right half of the table displays the results of the generalized estimation equation (GEE). The analysis did not show any correlation between one of the risk factors and a change or loss of acetabular correction (parameter estimates are within the upper and lower limits, *p* > 0.05, respectively)

### Radiographic management and follow-up

In the run-up to a TPO, every patient received a standardized antero-posterior (ap) pelvic radiograph in the radiological department of our institution. This image was used for deformity analysis and planning of the correction. The radiograph was produced in supine position, with a film-focus distance of 1,15 m; the beam centered between the symphysis and a line connecting the anterior superior iliac spines; both legs fully extended and 15° inwardly rotated. After fluoroscopically guided acetabular reorientation and osteosynthesis, at the very end of the surgery, an ap pelvic radiograph was performed on the operation table, technically executed as described above. All radiographs were scaled identically, for this reason, consistent metrical measurements were possible. The complete radiographic follow-up comprised pelvic radiographs 5 days, 6 and 12 weeks after the procedure (see above). All radiographs were performed in an identical manner in the radiological department of our hospital. The radiographs were archived in the picture archiving and communication system of our institution (PACS, GE Centricity Universal Viewer Version 6.0, General Electric Healthcare, Chalfont St Giles, UK). For each treated case, five pelvic radiographs were available for the assessment of acetabular orientation (preoperative, day of surgery, 5 days after surgery, 6 and 12 weeks after surgery). Consequently, in the postoperative follow-up, a change in acetabular correction was assessable for three different postoperative periods (see above). The radiographs of each case were checked for a relevant malrotation or tilt. Since in the female cases, the assessment of the distances from the symphysis to the tip of the coccyx or the sacrococcygeal joint was not possible due to the gonad shielding, we opted for a surrogate parameter: The width–height relation of the obturator foramen was used. To exclude an impact of the osteotomized pubic bone, the measurements were acquired on the side which was not operated (Fig. 1). A percentage variation of less than 10% was accepted. In this context, it should be emphasized that the radiographic parameters which were used in this examination (LCEA, AI, see below) have been proven to be particularly robust in the presence of a varying pelvic orientation [8].

The following exclusion criteria were defined: (1) severe deformation of the femoral head (e. g. after Legg-Calve-Perthes disease), (2) acetabular dysplasia as a part of a syndromic disease, (3) cases with an incomplete radiographic history (one or more postoperative images missing) and (4) cases with a percentage variation of more than 10% for the width–height relation of the obturator foramen.

### Measurement routine and definition of a change of acetabular correction

On each of the radiographs, three radiographic parameters were measured in order to assess acetabular orientation: lateral center edge angle (LCEA), acetabular index (AI) and the craniocaudal offset of the bony ends of the osteotomized superior pubic ramus (pubic osteotomy offset), resulting from the rotation of the acetabular fragment. After verification of the usability and the relevant landmarks, first of all, the center of the femoral head was estimated from a circle fit to its contour. Then, the longitudinal axis of the pelvis was defined by drawing a vertical line from the spinous process of L5 through the middle of the symphysis. The LCEA was measured between the line from the center of the femoral head to the lateral aspect of the sourcil and the longitudinal axis of the pelvis [9, 10]. AI was measured between a line connecting the inferior ischial tuberosities and a tangent to the most medial and most lateral aspect of the sourcil [11]. For the parameter pubic osteotomy offset, the craniocaudal offset of the pubic bone ends (cranial cortex), arising from the rotation of the acetabular fragment due to deformity correction, was measured metrically (millimeters (mm)) (Fig. 1). The three parameters were measured on all radiographs as described above by three observers at different levels of training (MK, BL, DD).

All three observers had to agree unanimously in order to define a change of acetabular correction. The observers assumed a change of acetabular correction based on their objectifiable measurements (differences of the values between the three intervals, see above) and subjective impression.

The change of acetabular correction was differentiated according to its magnitude and consequences: “slight change”: detectable but with no consequences in the aftercare regime; “relevant change”: entailing consequences in the aftercare regime (e. g. extended period of partial weight-bearing), “loss of correction”: entailing revision surgery.

### Risk factors

The patient records were reviewed with regard to potential risk factors for a change or loss of acetabular correction. The following risk factors were considered: age, gender, body weight, body height, body mass index (BMI), nicotine abuse, history of nicotine abuse, an incident of stumbling or falling in the first three months after surgery, wound healing disorder, classification according to the American Society of Anesthesiologists (ASA), severity of acetabular dysplasia (expressed by preoperative LCEA and AI) and a variation of the predominantly used fixation screw configuration (see above).

### Statistical analysis

An a priori power analysis was carried out for all statistical tests to calculate the needed sample size. The intra- and interobserver reliability was quantified with intraclass correlation coefficient (ICC) using a two-way model with agreement type. The values of ICC were interpreted according to the scale described by Cicchetti: less than 0.40: poor, between 0.40 and 0.60: fair, between 0.60 and 0.75: good and greater than 0.75: excellent [12]. For the analysis of risk factors, promoting a change or loss of acetabular correction, generalized estimating equation (GEE) was applied instead of generalized logistic regression in order to include the measurements of all three observers. A significance level of α = 0.05 was used for all tests. The statistical analysis was guided by a professional statistician and performed in “R” (version 4.2).

## Results

After application of the exclusion criteria, 225 hips were available for further examination (195 female hips, 30 male hips, age at the time of surgery 26.3 ± 8.5 years (mean, standard deviation (SD)) (please see the demographics in detail in Table 1). Correlation analysis with ICC resulted in excellent interobserver reliability between the three observers for almost all measured parameters and measurement dates (0.77–0.91). For the parameter, AI at the measurements 12 weeks after surgery good interobserver reliability (0.74) was calculated.

**Fig. 2:**
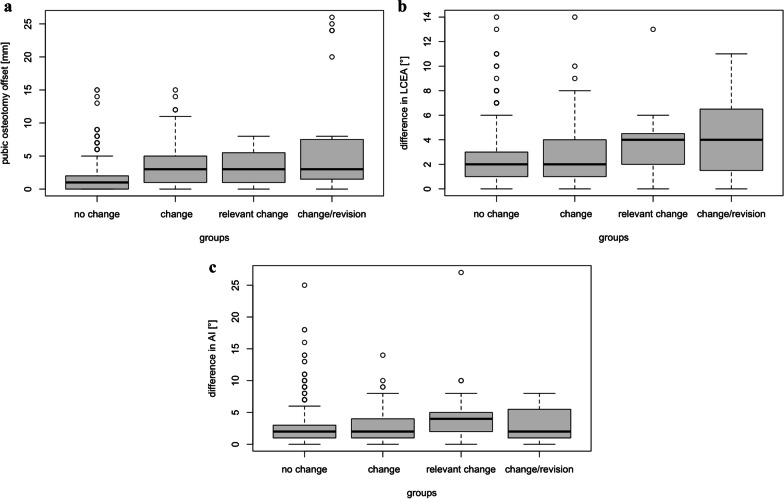
**a** The boxplots (representing from top to bottom: maximum, 1st quartile, median, 3rd quartile and minimum) display the analysis of the differences between two measurements (e. g. 5 days versus 6 weeks after TPO), considering the results of all three observers. From left to right: the left boxplot represents all measurements without a detectable change of correction (no change), the second from the left all changes of correction (change), the second from the right all relevant changes of correction (relevant change) and the right all changes with the necessity of revision (change and revision), respectively. For the detection of a rather slight change of acetabular correction, the parameter “pubic osteotomy offset” might have the best selectivity. **b, c** For the angle measurements (LCEA and AI), the selectivity might allow a differentiation between “no change” and “relevant change”

In 27 (12%) of 225 cases, the three observers agreed on a change of acetabular correction, based on the measured values and their subjective impression. In 18 (8%) of these, the change of correction was rather slight. In six cases (2.7%), the acetabular correction showed a “relevant change,” meaning that the it entailed consequences in the postoperative aftercare, e.g., a more restricted or prolonged partial weight-bearing. In three (1.3%) cases, the change in acetabular correction was not tolerable, meaning that revision surgery was recommended and performed. In these cases, a “loss of acetabular correction” was defined. For a detailed presentation of the angular and metrical measurements, please see Table 2 and Fig. 2 (Table 2; Fig. 2). The changes of acetabular correction were detected in each period of the postoperative course (Table 3).

**Table 2:** Differences of the measured values (mean, standard deviation SD) for the assessed parameters: lateral center edge angle (LCEA), acetabular index (AI) and the offset of pubic osteotomy

	Differences of all measurements (mean, SD)	Differences in changes of acetabular correction, total (mean, SD)	Differences in relevant changes of acetabular correction (mean, SD)	Differences in loss of acetabular correction (mean, SD)
Number of cases	2025	27	6	3
LCEA (°)	2.2 ± 2	3 ± 2	4 ± 3	4 ± 3
AI (°)	2.2 ± 2.2	3 ± 3	4 ± 4	3 ± 3
Offset of pubic osteotomy (mm)	1.9 ± 2.3	4 ± 4	5 ± 7	7 ± 9

The comparison of the mean differences of the angle measurements (LCEA and AI; interval 1–3, all 3 observers, n = 2025) and the mean differences, when a change in acetabular correction was defined (n = 27), do not allow a reliable differentiation based solely on these values. However, a difference of the values of 4° for LCEA and AI allowed an assumption of a “relevant change” or “loss of acetabular correction.” The offset of the pubic osteotomy might serve as a very useful parameter to detect a true change of correction. With this, differences in the radiographic imaging can be perceived with the naked eye. In addition, the metrical measurements might be more capable to distinguish a measurement inaccuracy and a true change of correction. In a “relevant change of acetabular correction,” consequences in the aftercare regimen were drawn, e. g. a more restricted or prolonged partial weight-bearing. In three cases, the change of acetabular correction was considered not tolerable. In these, the definition “loss of acetabular correction” was used. Revision surgery was performed

**Table 3:** Changes and losses of acetabular correction occurred in all postoperative periods

	Period 1 (day of surgery–5 days after surgery)	Period 2 (5 days after surgery–6 weeks after surgery)	Period 3 (6 weeks–12 weeks after surgery)	Total
Changes of acetabular correction (n)	15 (6.7%)	7 (3.1%)	5 (2.2%)	27 (12%)
Relevant changes of acetabular correction (n)	3 (1.3%)	2 (0.9%)	1 (0.4%)	6 (2.7%)
Loss of acetabular correction (n)	1 (0.4%)	1 (0.4%)	1 (0.4%)	3 (1.3%)

A slight tendency toward a change of correction in the first days after surgery was discernible

GEE did not show any correlation between the above-mentioned risk factors and a change or loss of acetabular correction (*p* = 0.79–0.99) (Table 1).

## Discussion

The two most important findings of this examination were: First, an unintended change or loss of acetabular correction cannot be attributed to specific risk factors. Second, the change or loss of acetabular correction occurred at different time periods after TPO.

In modern hip preservation surgery, the importance of precise acetabular orientation is emerging increasingly, since this has been proven to be crucial for the hip joint longevity [1–5]. Thus, it seemed obvious to us to investigate for risk factors and patterns of unintended changes in acetabular correction. In the current literature, there is very limited evidence about a change of acetabular correction in the early postoperative stage. In particular and to the best of our knowledge, there is no investigation looking at the dynamics of the acetabular fragment in a close-meshed follow-up in the first few months after pelvic osteotomy. Several authors reported on a loss of acetabular correction after PAO or TPO within the framework of a retrospective examination of general complications. Siebenrock et al. described a loss of correction in up to 4% in a series of 75 hip joints treated with PAO [13]. Clohisy et al. investigated a series of 17 severely dysplastic hip joints treated by PAO. In one hip joint in their series, an early loss of acetabular correction required open reduction and refixation. The authors reported that the patient affected by this complication started to consume two packs of cigarettes per day in the immediate postoperative period [14]. Katthagen et al. described the necessity of revision surgery in very few cases (less than 1%) in order to perform an improvement of the correction or to revise the osteosynthesis. The authors were able to overlook a rather large series of TPO with a total of 2036 procedures [15].

The present examination focused on potential dynamics of the acetabular fragment after TPO. In order to capture even subtle differences, each of the three observes performed 2700 single measurements of acetabular parameters (LCEA, AI, offset of pubic osteotomy) on the postoperative radiographs. For this analysis, a total of 900 pelvic radiographs were available, obtained in a highly standardized follow-up in the first 12 weeks after surgery. Based on their measurements and subjective impressions, the three observers agreed in 27 cases with the definition “change of acetabular correction.” The majority of these cases (18) showed a slight change. In six cases, the acetabular correction showed a “relevant change,” with consequences in the aftercare regimen. In three cases, a “loss of correction “ involved an intolerable change and entailed revision surgery. Of the three measured parameters, the “offset of pubic osteotomy” might have the best selectivity to distinguish a change in acetabular correction (4 ± 4 mm, 15 mm (mean, SD, maximum)) from a potential measurement inaccuracy (1.9 ± 2.3 mm (mean, SD)) (Table 2, Fig. 2a). For the angle measurements (LCEA, AI), the selectivity seems lower. The values might be helpful to detect a “relevant change” (LCEA: 4° ± 3°, 13°; AI: 4° ± 4°, 10° (mean, SD, maximum)) or “loss of correction” (LCEA: 4° ± 3°, 11°; AI: 3° ± 3°. 8° (mean, SD, maximum)) (Table 2, Figs. 2b, c).

Initially, the goal of this examination was to gather specific information with the view to align the postoperative follow-up regimen to individual risk factors. The postoperative care of our patients includes a closed-meshed protocol, as described above. On the assumption that all three observers would not have detected a change of acetabular correction, for example, in a female non-smoker with a normal BMI at the follow-up 6 weeks after surgery, this might have served as a scientific rationale to skip this follow-up. Contrary to our expectations, there was no significant statistical correlation between the defined risk factors and a change or loss of acetabular correction. A change or loss of correction had to be recognized even in patients without any risk factor. Additionally, the changes or losses of correction were not attributable to a specific period in the postoperative course. Indeed, there was a slight emphasis for a change of correction in the immediate postoperative period until the first follow-up 5 days after TPO, when the patients were mobilized for the first time. But the changes of correction also were observed in the period of partial weight-bearing, 5 days to 6 weeks after TPO and in the period of gradual load increase, 6–12 weeks after surgery. Based on our findings, this means that there is no rationale to skip a radiographic control and that every once treated patient has to be followed-up with the same attention, irrespective of the apparent risk profile or postoperative period. The vast majority of the rather slight changes of acetabular correction did not require any modification in the aftercare protocol. In six cases (2.7%), a relevant change of correction was recognized in time and the period of partial weight-bearing was extended. The acetabular correction remained acceptable. In three cases (1.3%), surgical revision was performed in order to restore the initial acetabular orientation. A major dislocation of the acetabular fragment did not occur. In our opinion, the results of this examination do not speak against a less restrained postoperative protocol as described elsewhere [16–18].

This examination has several limitations. First, it was not possible to clearly distinguish a change of acetabular correction from a measurement uncertainty, looking at the parameters alone (LCEA, AI and pubic osteotomy offset), since the discriminatory power did not allow this (Table 2). Apart from the measured values, a change of acetabular correction was defined in a mutual agreement of all three observers, additionally considering their subjective impression. Second, this examination focused on the dynamics of the acetabular fragment in the first 12 weeks after TPO, leaving the further clinical course outside. Hence, an impact on the clinical outcome or on the healing of the osteotomy sites cannot be foreseen [19–24]. Third, the predominately used pattern of acetabular fragment fixation (see above) had to be modified in some cases, e.g., when a crossing of the 4.5 mm steel screws in a thin iliac bone was not feasible without a collision of the implants. A variation of the screw configuration might have had an impact on the postoperative stability [7]. However, in this examination, there was no statistical correlation between a variation of the screw configuration and a change or loss of acetabular correction.

## Conclusion

In a highly standardized, closed-meshed radiographic follow-up of 225 hips treated with TPO, 8% of the hips showed a slight change of acetabular correction, detectable in the first 12 weeks after surgery. In 4% of the hips, a relevant change or loss of correction occurred and entailed consequences in the aftercare regimen. The measurement of the pubic osteotomy offset might be more sensitive to detect a slight change of acetabular correction than the measurements of LCEA and AI. The change or loss of acetabular correction could neither be attributed to a specific risk factor, nor to a certain period in the postoperative course. Based on the findings of this examination, there is no rationale to skip a radiographic follow-up in the first 12 weeks after TPO. We highly recommend to follow-up every once treated patient with the same attention, irrespective of the apparent risk profile.

## Data Availability

The authors agree to deposit the data that support the findings of this examination. The data have not been uploaded to a public repository yet.
